# Supplementary material for: Factors Influencing Breast Milk Antibody Titers during the Coronavirus Disease 2019 Pandemic: An Observational Study
Source: Nutrients. 2024 Jul 18;16(14):2320. doi: 10.3390/nu16142320 (PMC11280407; doi:10.3390/nu16142320)
Supplement: Supplementary file 1 [file nutrients-16-02320-s001.zip › Nutrients_Hochmayr_Supplementary Materials and Methods_final.pdf]

Supplementary Materials and Methods

Dried blood spot (DBS) anti-S1RBD-IgG and -IgM analyses

For analysis of whole blood anti-S1RBD-IgG and -IgM, samples obtained from umbilical cord blood and/or neonatal venous blood collected at 48 hours of life were blotted on Whatman filter paper (Whatman 903 protein saver card, Sigma-Aldrich Handels Gmbh, Vienna, Austria) and allowed to dry at room temperature for at least 3 hours. Dried blood spots (DBS) were stored in sterile plastic bags (VWR International, Vienna, Austria) with desiccant at room temperature until further processing. Elution of DBS samples for measurement of anti-S1RBD-IgG and -IgM concentrations was performed according to the manufacturer's instructions. In brief, two DBS punches (1x5 mm and 1x6 mm) per sample were immersed in 250 µl elution buffer and eluted on a shaker at 850 rpm at room temperature for 4 hours with additional vortexing for 10 seconds every 30 minutes. Afterwards, liquid without filter paper was transferred to a clean tube and centrifuged at 14000 rpm at room temperature for 10 minutes. Supernatant was collected in a fresh tube and stored at -20°C until further analysis. Eluted DBS samples were analysed using Raybiotech SARS-CoV-2-IgG and -IgM DBS ELISA (IEQ-CoVS1RBD-IgG-DBS and IEQ-CoVS1RBD-IgM-DBS Raybiotech, GA, USA). Samples were plated in duplicate for 1 hour. After incubation with anti-human IgG or IgM and HRP-Streptavidin for 30 minutes each, TMB solution was added to the plate for 15 minutes. Reaction was stopped with sulphuric acid and OD values were determined at 450 nm with a plate reader (Hidex Sense, HVD Life Science, Vienna, Austria). To calculate Ig concentrations, a positive control from DBS samples containing IgG and IgM against S1RBD protein (included in the kit) was employed to create a standard curve. In case two samples were available for one neonate, measured concentrations were averaged.

Three DBS samples from patients without SARS-CoV-2 infection or vaccination were used as negative controls. Samples exhibiting anti-S1RBD-IgG concentrations greater than 30 U/ml and anti-S1RBD-IgM concentrations greater than 100 U/ml were considered positive.
