# Supplementary material for: Factors Influencing Breast Milk Antibody Titers during the Coronavirus Disease 2019 Pandemic: An Observational Study
Source: Nutrients. 2024 Jul 18;16(14):2320. doi: 10.3390/nu16142320 (PMC11280407; doi:10.3390/nu16142320)
Supplement: Supplementary file 1 [file nutrients-16-02320-s001.zip › Nutrients_Hochmayr_Supplementary Table 1_final.pdf]

**Supplementary Table S1: Data availability according to breast milk sample type and anti-SARS-CoV-2-S1RBD-immunoglobulin (Ig) class.**

| Colostrum | IgA | IgG | IgM |
|---|---|---|---|
| available n (%) / missing n (%) | 111 (79.3) / 29 (20.7) | 107 (76.4) / 33 (23.6) | 107 (76.4) / 33 (23.6) |
| | | | |
| **Transitional milk** | **IgA** | **IgG** | **IgM** |
| available n (%) / missing n (%) | 82 (58.6) / 58 (41.4) | 82 (58.6) / 58 (41.4) | 82 (58.6) / 58 (41.4) |
| | | | |
| **Mature milk** | **IgA** | **IgG** | **IgM** |
| available n (%) / missing n (%) | 71 (50.7) / 69 (49.3) | 71 (50.7) / 69 (49.3) | 73 (52.1) / 67 (47.9) |

Data are presented as counts (n) and percentages (%).
